# Use of Mobile Technologies to Streamline Pretriage Patient Flow in the Emergency Department: Observational Usability Study

**DOI:** 10.2196/54642

**Published:** 2024-06-07

**Authors:** Panzhang Wang, Lei Yu, Tao Li, Liang Zhou, Xin Ma

**Affiliations:** 1 Department of Medical Informatics Shanghai Sixth People's Hospital Shanghai China; 2 Department of Orthopedics Shanghai Sixth People's Hospital Shanghai China

**Keywords:** overcrowding, overcrowded, crowding, smartphone, queueing, pretriage, self-service, triage, emergency, urgent, ambulatory, mHealth, mobile health, workflow, health care management, hospital

## Abstract

**Background:**

In emergency departments (EDs), triage nurses are under tremendous daily pressure to rapidly assess the acuity level of patients and log the collected information into computers. With self-service technologies, patients could complete data entry on their own, allowing nurses to focus on higher-order tasks. Kiosks are a popular working example of such self-service technologies; however, placing a sufficient number of unwieldy and fixed machines demands a spatial change in the greeting area and affects pretriage flow. Mobile technologies could offer a solution to these issues.

**Objective:**

The aim of this study was to investigate the use of mobile technologies to improve pretriage flow in EDs.

**Methods:**

The proposed stack of mobile technologies includes patient-carried smartphones and QR technology. The web address of the self-registration app is encoded into a QR code, which was posted directly outside the walk-in entrance to be seen by every ambulatory arrival. Registration is initiated immediately after patients or their proxies scan the code using their smartphones. Patients could complete data entry at any site on the way to the triage area. Upon completion, the result is saved locally on smartphones. At the triage area, the result is automatically decoded by a portable code reader and then loaded into the triage computer. This system was implemented in three busy metropolitan EDs in Shanghai, China. Both kiosks and smartphones were evaluated randomly while being used to direct pretriage patient flow. Data were collected during a 20-day period in each center. Timeliness and usability of medical students simulating ED arrivals were assessed with the After-Scenario Questionnaire. Usability was assessed by triage nurses with the Net Promoter Score (NPS). Observations made during system implementation were subject to qualitative thematic analysis.

**Results:**

Overall, 5928 of 8575 patients performed self-registration on kiosks, and 7330 of 8532 patients checked in on their smartphones. Referring effort was significantly reduced (43.7% vs 8.8%; *P*<.001) and mean pretriage waiting times were significantly reduced (4.4, SD 1.7 vs 2.9, SD 1.0 minutes; *P*<.001) with the use of smartphones compared to kiosks. There was a significant difference in mean usability scores for “ease of task completion” (4.4, SD 1.5 vs 6.7, SD 0.7; *P*<.001), “satisfaction with completion time” (4.5, SD 1.4 vs 6.8, SD 0.6; *P*<.001), and “satisfaction with support” (4.9, SD 1.9 vs 6.6, SD 1.2; *P*<.001). Triage nurses provided a higher NPS after implementation of mobile self-registration compared to the use of kiosks (13.3% vs 93.3%; *P*<.001). A modified queueing model was identified and qualitative findings were grouped by sequential steps.

**Conclusions:**

This study suggests patient-carried smartphones as a useful tool for ED self-registration. With increased usability and a tailored queueing model, the proposed system is expected to minimize pretriage waiting for patients in the ED.

## Introduction

For outpatients, registration at the hospital is completed online via appointments initiated by the patients themselves. However, for patients arriving at the emergency department (ED), registration is normally completed by triage nurses who communicate with patients and then interact with computers for data entry. Such nurse-led registration often takes time and slows down the triage process [1,2].

When a patient presenting at the ED is seen, others have to wait [2]. During peak hours, pretriage waiting tends to be prolonged and unpredictable [2-4], representing one of the main factors contributing to ED overcrowding [5,6]. An extended waiting time in the ED has been associated with increased morbidity and mortality rates [7,8].

Recently, based on the experience in other industries [9], there has been research interest in the use of self-service technologies (SSTs) such as kiosks [10] in the ED setting. For example, Sinha et al [11] demonstrated that the registration process can be easily completed by patients or their proxies using kiosks. However, unlike unmanned retail stores [12], there is no evidence that the use of kiosks can completely replace human-based triage (ie, triage nurses). Without proper control, full self-triage could lead to disparities. For instance, some younger and more computer-literate patients would easily jump ahead of the older, frail, less educated, and less computer-facile patients.

Using ED kiosks requires patients, nurses, and machines to work together. For example, self-awareness and path-finding are required for new arrivals. Otherwise, a referring service is required from triage staff [13], which directs the patient flow to a specific location to complete self-registration. Kiosks could break down at any time, which interrupts the work of the triage staff to handle technical failures [13]. In addition, triage staff need to monitor the kiosk area because long lines at the kiosk could delay patient identification [13].

The coworking context has an impact on the overall triage efficiency, and kiosks work well in offline EDs, but there are still some barriers to overcome for their use as an effective SST. Outside the ED setting, more and more online medical encounters are based on mobile SSTs (ie, smartphones) via social apps [14-18]. For example, smartphones could improve data quality by reducing registration errors [19]. In China, WeChat is the most popular social app, which has more than 1000 million monthly active users [20]. WeChat is not only a popular app but can also serve as a platform to host other web-based apps [21,22]. These apps are built into WeChat and do not need a separate installation on patient smartphones. The apps can even be opened directly when the owner scans an eligible QR code. Beyond medical encounters, a mobile SST is also widely used in the context of mobile payment [23,24]. During COVID-19, the Chinese government used QR technologies to engage its citizens to fight the pandemic [25,26].

For security reasons, patient smartphones cannot be connected to ED triage servers, which limits the exploration of mobile SSTs in EDs. In a previous study, we used a tailored security architecture to send patient-reported data from patients’ smartphones to hospital networks via an offline QR code reader [27]. Subsequently, Song et al [28] used a similar approach to transfer medical data to hospital information systems. In this study, we expanded on this work to explore a self-registration tool based on the stack of QR technologies and WeChat. To measure the impact on pretriage patient flow, we compared the use of mobile self-registration with the use of kiosks in a real-world setting. Our goal was to investigate the use of mobile technologies to improve pretriage flow in EDs.

## Methods

### Rationale

In March 2020, kiosks were introduced at a busy metropolitan ED in Shanghai with an annual census of 306,000 patients. In the crowded greeting area, the ED set up four kiosks to direct nonambulance arrivals for self-registration. With increasing uptake, lines to access the kiosk were frequently observed, prompting development of an alternative method.

### Coworking Flow

**Figure 1:**
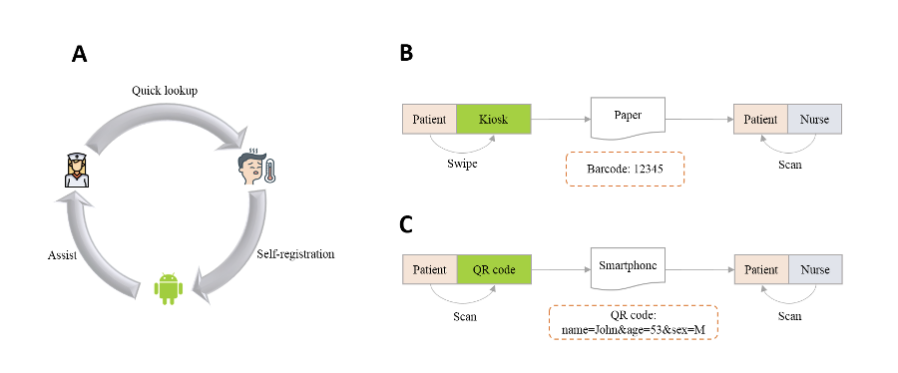
Coworking context for self-registration at the emergency department. (A) The roles in the coworking context. (B) The kiosk-led pretriage flow. (C) The smartphone-led pretriage flow.

This study was inspired by the concept of industry 5.0 [29]. With the increasing use of SSTs, the roles of nurses, patients, and machines were redefined in a coworking context (Figure 1A). Some repeated tasks shifted from nurses to tools and patients. Electronic forms were used to replace paper-based forms. The tool guides patients to complete some paperwork that was previously conducted by triage staff.

A patient needs to travel from the front end to the back end of the system, whether via kiosks (Figure 1B) or via smartphones (Figure 1C). The workflow was broken down into five different steps (Table 1). For kiosks, patients swipe their health cards on the front end for registration and present a paper document with a barcode to a triage nurse. After scanning the code with a hand-held device, the nurse quickly finds the record and then updates it with an acuity level based on the “quick-lookup” result [30-32].

For smartphone users, WeChat is used to scan a QR code at the front end. After filling out the form, the result is encoded into a QR code on patient smartphones using a structured format (Figure 1C). Patients show the digital proof to a triage nurse at the back end. The nurse scans the QR code and automatically creates a record after extracting the structured content. The nurse then uses the “quick-lookup” tool to assign an acuity level for patient triage.

**Table 1:** Comparison of the workflow in self-registration at the emergency department using a kiosk and a smartphone.

Step	Kiosk	Smartphone
Reach	With or without a referral, a patient walks a certain distance to the kiosk area and finds an available machine for registration	With or without a referral, a patient scans a QR code posted along the route using WeChat. A web-based registration page is then displayed and they need to find a site nearby for registration
Initiate	A patient navigates to the homepage to find the launching icon and swipes a card for log-in or manually inputs the information when the card is not on hand or card-swiping fails	The patient enters the associated phone number to start
Input	On the public touchscreen	On the patient’s smartphone
Print	The result is printed into a paper document	The result is printed into a QR code on the smartphone
Submit	The patient brings the printed paper result to the registration desk. The triage nurse uses a personal digital assistant equipped with a scanner to read the barcode.	The patient brings the smartphone to the registration desk. The triage nurse uses a personal digital assistant equipped with a scanner to read the QR code. A short confirmation message is sent to the patient via their registered contact number

### Self-Registration Content

For registration, patients have to enter some personal information and the presenting reason for the ED visit (Table 2). With autocomplete-based suggestions for presenting problems [19], the system helps patients enter their chief complaints more efficiently and accurately. For kiosks, swiping a card is useful for saving time by avoiding having to input repeated data. However, when the card is not on hand, for security reasons, the patient has to enter all of the information manually. For kiosk users, the contact number is optional, thereby relying on triage nurses for patient validation. For smartphone users, the contact number is mandatory. When the content is scanned into the ED system by the triage staff, a confirmation message is sent to the patient according to the contact number. For experimental purposes, patients were required to fill out a field to indicate whether self-registration followed a referring service from clinical staff.

Technically, it is possible to use self-triage algorithms to assign an acuity level for a given patient condition [10]. However, this might lead to queue-jumping when the evaluation process is controlled by the patients themselves. In this study, the system was set up so that the evaluation result was not immediately available to patients after self-registration, but rather appeared as a suggestion to the triage staff on the hand-held screen. The triage nurse had the right to modify the result after human-based evaluation. The whole process was designed to mitigate paperwork burdens for triage staff.

**Table 2:** Content of the registration form.

Element code	Element description
Phone	Contact telephone number
Name	Patient name
Sex	Patient sex
Age	Patient age
Race	Patient race
Complaints	A list of standardized codes for chief complaints, separated by commas
Reason	Patient narrative text to explain the reason for presenting to the emergency department
Referred	Using the self-registration tool after being referred by an assistant

### Security Architecture

For kiosks, the platform and the collected data were completely hosted in the hospital private network. Attacks from the internet were handled by the hospital firewall.

For smartphones, the front-end registration page was completely disconnected from the back end. Technically, this was designed as a static webpage hosted by the WeChat platform, which rendered a web form to allow patients to complete the registration online. However, the platform handled data submission in a different manner. Instead of transmitting the collected data over the internet, the data were saved to the local storage on the patient’s smartphone, being displayed as a QR result. The QR result could then be read via the scanner held by the triage nurse. Thus, the data flow was executed from online to offline in a smart and secure manner. Since no third-party servers are involved, data security and privacy can be well protected. Hosting the static webpage on a mature platform with WeChat had benefits for both availability and scalability.

### Multilingual Support

As a public machine, it is difficult for a kiosk to determine the language preference of an upcoming user. Therefore, a button for language switching was installed on the public screen. By contrast, for smartphone users, the system can automatically detect the language preference based on the location setting.

### Signage

**Figure 2:**
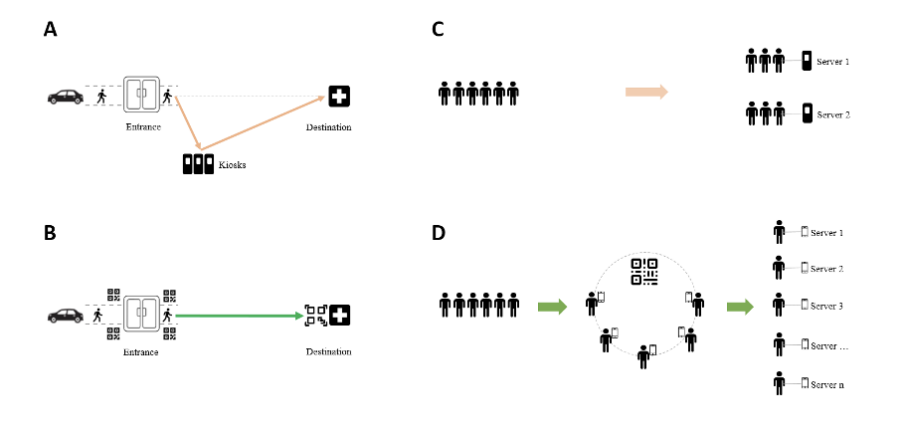
Signage and queueing. (A) Signage and patient flow in the kiosk group. (B) Signage and patient flow in the smartphone group. (C) Queues in the kiosk group. (D) Queues in the smartphone group.

Based on best practice [13,33], the kiosk area was placed inside the ED (Figure 2A), visible from the registration desk. Roadmaps were provided. For smartphones, the signage was printed out as QR symbols, which were posted in four different places, inside and outside the entrance (Figure 2B). The size of the signage was appropriately determined so that it could be easily scanned within a distance (Figure 2D). These QR codes encoded a link to the self-service app.

### Study Design

The evaluation was performed during peak hours from 5 PM to 9 PM in three EDs. In each center, an assistant was recruited to work near the entrance to make sure patients traveled to the service quickly by offering a referral service. Patients were told they could bypass self-registration and go directly to the registration desk. Both patients and their proxies were allowed to use the self-registration tools. Data were collected during a 20-day period in each center. Random days were chosen for the use of kiosks and smartphones. At a given time, only one digital tool was evaluated to direct the patient flow. For example, when kiosks were evaluated, the signs with QR codes were removed from the entrance.

In each center, assistants (N=4) were recruited to work at the self-service area to assist patients for self-registration. All of the assistants had a background in qualitative research, and they were trained on how to handle technical issues related to self-registration that could result in triage interruptions [34]. Their observations were documented at the end of each day. Two authors (TL and LY) with expertise in informatics, human factors, quality and safety, and qualitative research served as coders and reviewers. These authors worked with the assistants for coding. Once coding was complete, thematic analysis [35] was used to group codes. Discrepancies were discussed by both the coders and the assistants and reconciled by consensus prior to final analysis.

To evaluate timeliness and usability, medical students (N=210) were recruited to simulate ED arrivals. They received no training and were asked to arrive randomly. Upon arrival, the timestamp (*T_arrive_*) was recorded. When they received an acuity result from triage staff, the timestamp, or *T_end_*, was recorded. The interval (*T_end_*–*T_arrive_*) was used to denote pretriage waiting times. For usability evaluation, the After-Scenario Questionnaire (ASQ) [36] was used, which is a standard questionnaire with three questions: “ease of task completion,” “satisfaction with completion time,” and “satisfaction with support.” The ASQ has been validated and is commonly used in studies related to mobile health [37]; this tool is particularly suitable in scenarios where a user might finish a task despite not successfully completing the task. Scale options were set according to a range of 1 to 7, with higher scores representing a higher degree of satisfaction. Two additional assistants were recruited to work in certain posttriage areas to administer paper-based ASQs. An unanswered question was treated in the same manner as a “not applicable” (NA) question. Since the study was designed to evaluate self-services, most participants might complete a session without any help, leaving the question of “satisfaction with support” unanswered. However, since “satisfaction with support” was designed to measure the level of self-service received, these “NA” answers were treated as the highest score in this study. For “ease of task completion” and “satisfaction with completion time,” records with “NA” were treated as invalid feedback.

During the evaluation, triage nurses (N=30) were recruited to work on a shift in each group and two nurses were assigned to the triage unit on each shift. The Net Promoter Score (NPS) [38] was used to solicit the nurses’ opinions in using the tool to handle patient flow, including questions such as “On a scale from 0 to 10, how likely are you to recommend this tool to ED managers to deal with pretriage patient flow?” Participants scoring 9 or 10 were classified as “promoters,” those providing a rating of 7 or 8 were classified as “passives,” and those providing a rating from 0 to 6 were classified as “detractors.” The final NPS was calculated by subtracting the percentage of detractors from the percentage of promoters (excluding the passives). The result could range between –100% and +100%, and the performance was marked as “good” for scores above 50% [39].

### Statistical Analysis

Data analysis was performed using SPSS software (version 27.0). The *χ*^2^ test was used to analyze proportion data. All analyses were based on a two-sided *P* value, with *P*<.05 considered statistically significant.

### Ethical Considerations

According to regulations of the Shanghai Ethics Committee for Clinical Research [40], the requirement for ethical approval was exempt since the hospital only exports deidentified data for study purposes. Participants were informed about the study orally. Individual and privacy-related data were not used in this study.

## Results

### Primary Results

**Figure 3:**
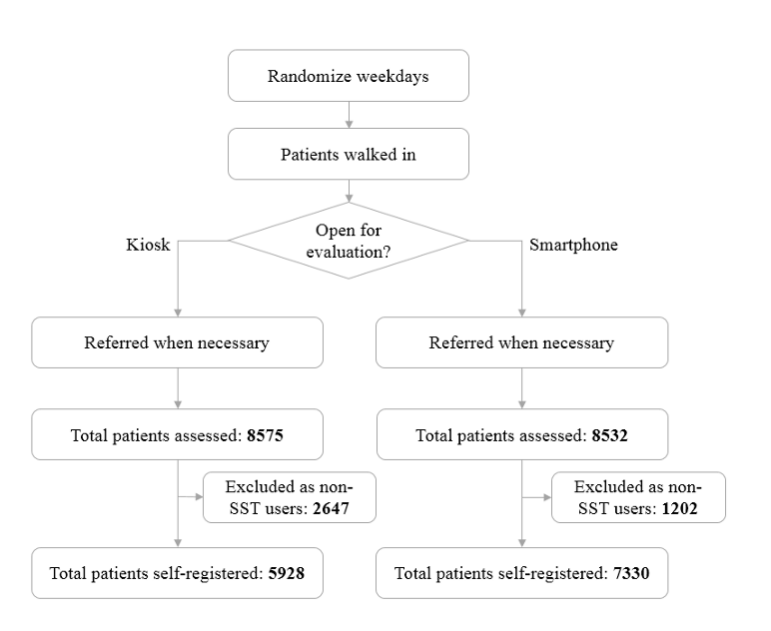
Recruitment flowchart. SST: self-service technology.

The recruitment flow of real patients is displayed in Figure 3 and the participant characteristics are summarized in Table 3. Within the study time frame, 5928 of 8575 patients (69.13%) performed self-registration on kiosks and 7330 of 8532 patients (85.91%) checked in on their smartphones, representing a significant difference (*P*<.001). There was also a statistically significant difference in the percentage of patients referred to kiosks and smartphone self-registration (Table 3).

Timeliness and usability were evaluated using simulated arrivals (N=210) in each group. Compared to kiosks, pretriage waiting times were significantly reduced in the smartphone group, whereas the smartphone group scored significantly higher in the usability items “ease of task completion,” “satisfaction with completion time,” and “satisfaction with support” (Table 3).

The NPS was calculated based on feedback obtained from triage nurses (N=30) in each group. According to their scores, there were 14 promoters, 6 passives, and 10 detractors in the kiosk group, whereas there were 28 promoters, 2 passives, and 0 detractors in the smartphone group. The use of smartphones significantly improved the final NPS compared to the use of kiosks (Table 3).

**Table 3:** Comparison of baseline characteristics across groups.

Characteristics			Kiosk		Smartphone		*P* value
**Real patients**							
	Total patients assessed, n	8575		8532		—^a^	
	Self-registrations, n (%)	5928 (69.1)		7330 (85.9)		<.001	
	Age (years), mean (SD)	42.1 (21.1)		43.1 (20.6)		.91	
	Male sex, n (%)	2727 (46.0)		3291 (44.9)		.20	
	Sessions completed with referring, n (%)	2591 (43.7)		645 (8.8)		.001	
**Simulated patients**							
	Participants, n	210		210		—	
	Age (years), mean (SD)	22.5 (3.2)		22.6 (2.9)		.75	
	Male sex, n (%)	106 (50.5)		101 (48.1)		.63	
	Pretriage waiting time (minutes), mean (SD)	4.4 (1.7)		2.9 (1.0)		.001	
	Ease of task completion, mean (SD)	4.4 (1.5)		6.7 (0.7)		.001	
	Satisfaction with completion time, mean (SD)	4.5 (1.4)		6.8 (0.6)		.001	
	Satisfaction with support, mean (SD)	4.9 (1.9)		6.6 (1.2)		.001	
**Triage nurses**							
	Participants, n	30		30		—	
	Age (years), mean (SD)	36.8 (9.3)		36.0 (8.8)		.73	
	Male sex, n (%)	3 (10)		4 (13.3)		.69	
	Promoters, n	14		28		—	
	Passives, n	6		2		—	
	Detractors, n	10		0		—	
	NPS^b^, %	13.3		93.3		.001	

^a^Not applicable.

^b^NPS: Net Promoter Score.

### Patient Flow

**Figure 4:**
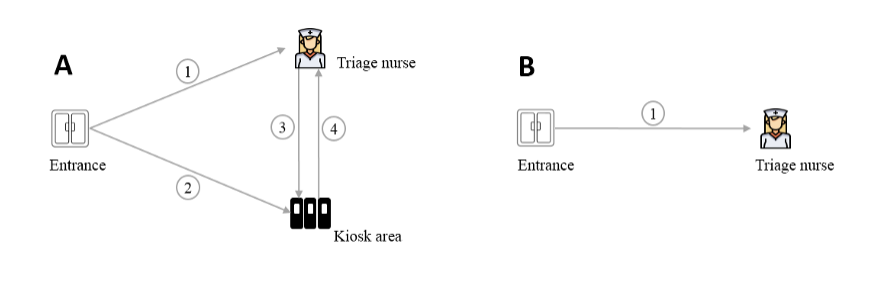
Paths in the pretriage flow. (A) Patient flow in the kiosk group. (B) Patient flow in the smartphone group.

While kiosk users competed with each other while waiting in lines (Figure 2C) for check-in, smartphone users encircled (Figure 2D) a posted QR symbol to start using the tool. After scanning, smartphone users quickly dispersed around the ED to use their devices privately; thus, no queues were formed in the smartphone group. As illustrated in Figure 4, there were 4 paths in the kiosk group: (1) patients skipped self-registration and walked directly to triage staff, (2) patients walked to the kiosk area for self-registration, (3) patients left the kiosk area to see triage staff, and (4) patients switched to the self-registration line after arriving at the traditional registration desk. By comparison, there was only 1 path in the smartphone group: all patients followed the same path for self-registration and triage.

### Reach

Compared to the kiosk area, the posted QR symbols appeared to be easily seen by arrivals and then resulted in followers. Assistant B4 described that “It is good for patients to find and follow, especially posted outside.” By contrast, in relation to kiosks, the assistant indicated that “the roadmap seems not working that well.”

Although patients waited to be triaged in no particular order, the crowded situation in front of the registration desk could be seen from a distance. In the kiosk area, patients had to wait in order of their arrival, and the lines were also easily seen from the triage area. The two endpoints attracted patients and sometimes resulted in extra traffic of switching. Assistant A2 commented: “It is fine but I have a concern for patient safety when these two lines compete for patients.” This concern was not found in the smartphone group.

Patient proxies were allowed to perform the actual data entry. However, in the kiosk group, the proxy often stepped into the line for the kiosk with the patient, resulting in a more crowded area. Although this is helpful for conversation-based data entry, it definitely increased the density of the self-service area. Indeed, assistant B2 advocated that “queue discipline is a must for kiosks.” For smartphones, the proxy simply went ahead with scanning the QR code and then returned to the patient in an open area for collaborative data entry. The assistant then concluded, “the queue disappeared and it’s privacy-friendly.”

A kiosk might break down at any time. When this occurs, the clinical staff should be notified in a timely manner so that an “under repair” sign can be set up. However, this was not often the case in practice; instead, as described by assistant C3, “some patients just switched to another line or simply quit.” In the case of smartphones, most interruptions were related to a low battery or bad signal. A smartphone failure would not cause a delay for other patients.

### Initiate

Kiosks can be used not only for self-registration but also for other purposes [33], and each case requires its own launching icon on the homepage. Patients faced burdens with respect to page navigation and language switching. For example, the previous session might not end as expected, which would prevent appropriately resetting the screen to the homepage.

Card swiping was considered to be convenient but was also associated with intermittent failures such as an inability to read the card or swiping on the wrong side. Manual input was provided in case of any failure. However, this required some cognition effort, as described by assistant A1: “some patients spent too much time on trying.” Patients who forgot their cards could also use the manual input option; however, this caused some issues, as described by assistant B3: “some patients blocked the line and stood too much time in front of a kiosk for searching.”

By comparison, it was more straightforward for smartphone users to get started. After scanning, the page showed up right away without requiring any additional information to be input such as a password.

### Input

During data entry, a kiosk failure was so disruptive that the entire line was impacted. Assistant C2 logged the following: “the entire line was moved to another.” No failures were logged for smartphones in this step.

### Print

After data entry was complete, the record was materialized as a proof. Namely, kiosks generated a paper document and smartphones used a QR code. With this proof, patients could confirm with the triage nurse that self-registration had been completed. However, patients had to spend some time to tear the paper document carefully from the printer. In some cases, the workflow was paused due to jammed paper, partially torn paper, or the printer being out of paper or ink. These delays were disruptive, as reported by assistant C1: “it really takes time to recover and most patients cannot wait.” By contrast, printing QR codes on smartphones was instant and no failures were reported.

### Submit

As mentioned above, patients needed to tear off the paper document from the printer before leaving the kiosk area. However, there were some cases in which more than one document was left in the printer. Consequently, some patients took the wrong record for submission, as logged by assistant A3: “Today, a record was marked with a wrong acuity level. It turned out that the patient took a wrong document.” Such an out-of-sequence issue was never found in the smartphone group.

In the kiosk group, some printouts were occasionally found to be left on the floor. No such data privacy issues were observed in the smartphone group.

## Discussion

### Principal Findings

ED overcrowding is an unresolved issue worldwide [5,41], which threatens patient safety and public health [42]. Therefore, it is an urgent need to address the long pretriage waiting time, as it is associated with an increased level of morbidity and mortality [7,8]. Patient registration becomes a bottleneck when data entry is completed by triage nurses. With SSTs, triage nurses can focus on higher-order tasks [10,43] and enable rapid assessment [44] in EDs.

Unlike outpatients, registration is not a prerequisite for ED patients. This study thus contributes to improving ED self-registration in two ways. The first is related to the actual device provider. Instead of using in-house devices for registration, patients can use their own smartphones with advantages of flexibility and scalability. The second contribution is related to improved digital interaction. “Scan-to-process” should be easy for nurses, as this is already a ubiquitous technique in medical settings (eg, barcode-based medication administration) [45]. During COVID-19, QR codes were widely used for contact tracing [46,47]. Therefore, visitors are now familiar with the rule of “scan to enter” before stepping into public buildings. Accordingly, we applied this concept for patients to adapt to the new workflow of ED check-in.

EDs are space-constrained areas [48] and thus patient flow occurs in an often crowded setting. In addition to the registration desk, the use of a self-registration kiosk introduces another point of convergence. Kiosks cannot be positioned too close together in the case of a sudden increase in arrivals. Theoretically, one flow will start at the ED entrance, pass through to the kiosks, and finally meet up with triage nurses, while the other flow is directly from the entrance to the triage area. In reality, there may be a third path in which patients might return to the kiosk area for registration from the line formed at the registration desk to see a human after balancing out the potential waiting times. Passing through kiosks is helpful for reducing triage burden, but the increased movement within the ED and midway congestion might be detrimental to patient safety [49], especially for patients with potential critical illnesses. Smartphones barely have an impact on the incoming flow. Outside the entrance, scanning takes only a few seconds, and then patients can walk into the ED and complete registration while waiting to be triaged in front of nurses. In this way, experienced nurses could have a better chance of quickly scanning the patients to determine who should be treated first, even if the registration is not complete.

Although there was not a substantial difference in terms of completion time, using smartphones for self-registration could completely eliminate the prominent waiting issue caused by limited resources in EDs, as it enables an infinite-server queueing model [50]. Thus, serviceability and scalability could be refined from a different lens.

Usability is a key factor for users’ continuance intention. Some patients who have experienced kiosk malfunctions or delayed help may not use the kiosk area the next time they have to visit the ED. This can explain the low number of participants in the kiosk group. In China, WeChat and QR technologies are frequently used [20]; therefore, patients will be familiar with a QR-based interaction when approaching the ED entrance. Smartphone users also encounter few failures during the process, which might enhance their willingness to continue using the mobile check-in service.

Indeed, the triage nurses gave the smartphones a higher usability score than the kiosks. There could be several reasons for this difference. First, kiosks generate more interruptions than smartphones, which is supported by the ASQ results and observations. Triage staff have to pause their work to handle any kiosk-related interruptions. Second, the overall observation could have an impact, as arrivals are directed in a flow for self-registration and triage. Third, with respect to the signage, the kiosk area is more challenging as a prerequisite for registration compared to the ED entrance, resulting in high referring effort. Finally, smartphones could cause less delay for patient identification than kiosks.

Smartphones increase hospitality in the pretriage flow [51]. First, compared to public screens, smartphones honor patient privacy. Second, the use of personal devices ensures hygiene and cleanliness, which are more difficult to maintain when using shared kiosk touchscreens. Third, social distancing is important in the ED, which could pose a challenge when lining up at kiosks to compete for timely identification. Finally, smartphones are more user-friendly in multilingual scenarios.

Although smartphones have numerous benefits, a computer-literate arrival can still choose to use a kiosk when a smartphone is not at hand. In addition, those who are not comfortable with SSTs can still register at the human-based line. Thus, providing numerous options in EDs can help to improve triage efficiency, especially during peak hours.

### Limitations

Our study has limitations. First, this study was performed in three metropolitan ED centers in Shanghai; hence, our findings do not represent other disparate geographical areas with different ED volumes. For example, scanning QR codes might not be a common behavior adopted by patients in other countries. Second, due to the limited time frame of observation, some issues might not have been disclosed. Third, the smartphone-based tool was developed for the purpose of this evaluation and more features should be added in the future, such as integrating the tool with mobile sensors and using algorithms to predict triage results for decision-making. The lack of such features may have affected the usability results but could also serve as a basis to measure future enhancements.

### Conclusions

EDs are overcrowded with long waiting times for registration and triage. Patient registration is managed in a single thread when completed by a triage team. Therefore, using SSTs would ease the burden on triage nurses and allow them to focus on higher-order tasks. Compared to kiosks, smartphones seem to be more convenient and suitable as a pretriage SST. However, it is recommended to offer multiple options of self-registration services in EDs.

## Abbreviations

ASQ: After-Scenario Questionnaire
ED: emergency department
NA: not applicable
NPS: Net Promoter Score
SST: self-service technology

## Data Availability

The data sets used or analyzed during this study are available from the corresponding author upon reasonable request.
